# PigFRIS: A Three-Stage Pipeline for Fence Occlusion Segmentation, GAN-Based Pig Face Inpainting, and Efficient Pig Face Recognition

**DOI:** 10.3390/ani15070978

**Published:** 2025-03-28

**Authors:** Ruihan Ma, Seyeon Chung, Sangcheol Kim, Hyongsuk Kim

**Affiliations:** 1 Department of Electronics and Information Engineering, Jeonbuk National University, Jeonju 54896, Republic of Korea; 2 Core Research Institute of Intelligent Robots, Jeonbuk National University, Jeonju 54896, Republic of Korea

**Keywords:** deep learning, smart pig farm, pig face recognition, smart farm, face inpainting, fence occlusion segmentation

## Abstract

Accurate animal face recognition is essential for effective health monitoring, behavior analysis, and productivity management in smart farming. However, environmental obstructions and animal behaviors complicate identification tasks. In pig farming, fences and frequent movements often occlude essential facial features, while high inter-class similarity makes distinguishing individuals even more challenging. To address these issues, we introduce the Pig Face Recognition and Inpainting System (PigFRIS). This integrated framework enhances recognition accuracy by removing occlusions and restoring missing facial features. PigFRIS employs state-of-the-art occlusion detection with the YOLOv11 segmentation model, a GAN-based inpainting reconstruction module using AOT-GAN, and a lightweight recognition module tailored for pig face classification. In doing so, our system detects occlusions, reconstructs obscured regions, and emphasizes key facial features, thereby improving overall performance. Experimental results validate the effectiveness of PigFRIS. For instance, YOLO11l achieves a recall of 94.92% and a ${\mathrm{AP}}_{50}$ of 96.28% for occlusion detection, AOTGAN records a FID of 51.48 and an SSIM of 91.50% for image restoration, and EfficientNet-B2 attains an accuracy of 91.62% with an F1 Score of 91.44% in classification. Additionally, heatmap analysis reveals that the system successfully focuses on relevant facial features rather than irrelevant occlusions, enhancing classification reliability. This work offers a novel and practical solution for animal face recognition in smart farming. It overcomes the limitations of existing methods and contributes to more effective livestock management and advancements in agricultural technology.

## Simple Summary

Accurate pig identification is essential in smart farming, yet fences and other obstructions often block critical facial features, significantly reducing recognition accuracy—especially given the high similarity among individual pigs. To address these challenges, we propose a three-stage Pig Face Recognition and Inpainting System (PigFRIS), which integrates a Fence Occlusion Segmentation module (YOLOv11L), a GAN-based inpainting model (AOT-GAN) to restore occluded regions, and an EfficientNet-B2 recognition component for robust pig classification. By precisely detecting and correcting missing features, PigFRIS shifts the focus from incidental cues to the pig’s genuine facial attributes, delivering reliable recognition even in highly obstructed farm environments.

## 1. Introduction

Occlusions have emerged as a key obstacle to reliable pig face recognition, particularly under real-world farm conditions where metal fences, bars, or gating structures frequently obscure the most discriminative facial features [1,2]. Eyes, snouts, and ears—essential for distinguishing one pig from another—are often partially or fully blocked, thereby causing substantial drops in identification accuracy. In commercial barns, this issue is further compounded by the inherently high facial similarity among pigs, which already makes them challenging to differentiate. As a result, fence-induced occlusions have become one of the most pressing hurdles to the practical application of non-invasive pig face recognition systems, preventing otherwise promising deep learning models from achieving consistent performance.

While traditional livestock identification methods such as ear tags, tattoos, or RFID may circumvent the occlusion problem by relying on physical markers, they often entail significant labor costs, can be intrusive, and may fail when devices are lost or damaged [3,4]. Moreover, such methods do not leverage the benefits of automated, camera-based monitoring—an approach that has gained traction in smart farming for reducing manual intervention and improving animal welfare [5]. Motivated by these limitations, non-invasive approaches have gained increasing traction. Biometric systems using facial recognition offer a promising alternative that can improve both animal welfare and operational efficiency. Deep learning methods have been successfully applied to facial recognition in various species, including cattle [6,7], sheep [8], dogs [9], and birds [10]. Pig face recognition research has likewise advanced, with Hansen et al. [11] achieving around 83% accuracy using Convolutional Neural Networks, and Marsot et al. [12] noting that focusing on features like the eyes and snout can boost accuracy to about 91%. Recent works, including those of Li [13] and Shi [14], further demonstrated the potential of deep learning techniques in achieving rapid and robust pig face detection under practical farm conditions. Moreover, lightweight models also show promise for real-time farm applications [15]. Yet, these models often underperform when confronted with large-scale, systematic occlusions from fences or other barn structures. In one notable study, Liu et al. found that accuracies dropped from 94% to 81% when fences obstructed key areas of the pig’s face [16].

Various methods have been explored to address the occlusion problem in pig face recognition, loosely divided into two categories. The first involves occlusion-robust feature extraction, where models are either trained with augmented datasets that include occlusions or are designed with specialized attention mechanisms. These techniques can offer some resilience but often assume that the occluded regions are random or small in area, which may not hold when metal bars systematically block large portions of the face. The second category is image inpainting, a field that has advanced considerably since the introduction of Generative Adversarial Networks (GANs) [17]. GAN-based inpainting has proven adept at restoring missing or damaged pixels in various contexts [18,19], yet generic solutions trained on diverse datasets can struggle to reproduce the subtle textures and geometries of pig faces, especially when large segments of the face are fenced off. The shape of the bars themselves—often linear, repetitive, and covering critical features—further complicates generic inpainting pipelines, which may fill in occlusions with visually plausible yet identity-irrelevant content.

A direct response to these challenges requires a system that explicitly detects fences, recovers occluded areas with pig-specific details, and then leverages the restored images for recognition. Motivated by this need, we propose an integrated three-stage framework called the Pig Face Recognition and Inpainting System (PigFRIS). Rather than relying on partial solutions that either attempt to learn occlusion-robust features or apply generic inpainting, PigFRIS unifies fence detection, targeted GAN-based restoration, and a lightweight classifier into a single pipeline. By localizing the fence occlusions first, the method can precisely identify which image regions need to be reconstructed, allowing a specialized inpainting algorithm to focus its generative capacity on reproducing core features vital for identification. Subsequently, a resource-friendly classification model processes these refined images, achieving substantially improved accuracy under realistic farm conditions compared to using raw, occlusion-riddled inputs.

In PigFRIS, the first stage of this pipeline is the Fence Occlusion Segmentation module, which isolates metal bars and other structural obstructions that block critical facial features. We employ a supervised segmentation approach based on a customized YOLOv11L model [20] and a dataset of pig face images that are naturally occluded by fences in various orientations and lighting conditions. Human annotators use pixel-level labeling tools to precisely outline the fence regions in each image, thereby teaching the model to differentiate barred occlusions from the pig’s facial areas. Once trained, the model automatically generates accurate occlusion masks for newly acquired images, providing essential guidance for the inpainting process that follows.

After the occlusion masks are extracted, the second stage—the Pig Face Inpainting module—relies on an Aggregated Contextual Transformations GAN (AOT-GAN) [21] that is fine-tuned to the texture and geometry of pig faces. This component similarly requires a supervised framework, meaning it learns to recover the missing facial regions based on pairs of “clean” and “masked” images. To build such training data, we again utilize non-occluded pig faces as ground truth and systematically overlay simulated fence masks to generate the source images. During training, the GAN sees both the masked input (with fence-induced occlusions) and the corresponding unmasked face, thereby acquiring the ability to fill in realistic details for eyes, snouts, and other salient markings. This arrangement ensures that the model, once trained, can seamlessly replace occluded areas with plausible pig facial features. The inpainting process is especially critical in preserving subtle distinctions among pigs, given that even minor inaccuracies around the eyes or snout can degrade recognition performance.

In the final stage, the Pig Face Recognition module utilizes an EfficientNet-B2 classifier [22] to identify individual pigs from both inpainted and original images. We opt for this lightweight model in anticipation of real farm environments, where computational resources may be constrained but rapid inference is desired. The improved clarity delivered by the occlusion segmentation and GAN-based inpainting steps markedly enhances recognition accuracy compared to using raw, occlusion-riddled images alone. Empirical observations show that when fence occlusions remain uncorrected, recognition accuracy drops significantly, underscoring how severely obstructed faces impair model performance. By contrast, once PigFRIS has automatically removed fence regions and reconstructed the missing facial features, the identification rates increase notably. The system therefore offers a practical solution to the long-standing limitation of occluded pig faces, promising non-invasive animal identification in large-scale operations and contributing to the broader adoption of smart farming practices.

In practical on-farm deployment, the Pig Face Recognition and Inpainting System (PigFRIS) is designed to process incoming video streams or image feeds in near real-time, addressing fence occlusions on the fly. As the camera captures pig faces, the Fence Occlusion Segmentation module first detects the bars or other obstructing structures using the trained YOLOv11L-based segmentation network. Whenever an occlusion mask is identified, the system immediately feeds the masked pig face into the Pig Face Inpainting module, which employs the fine-tuned AOT-GAN to reconstruct the concealed facial regions. Once the missing areas are realistically restored, the resulting inpainted image is passed to the EfficientNet-B2 classifier for final identification. By chaining these three stages together in a continuous loop, PigFRIS ensures that obstructed pig faces are accurately processed and recognized as they appear in real-time, offering a practical solution that aligns with the rapid pace of modern farm operations.

- We propose an integrated three-stage system (PigFRIS) that systematically addresses fence occlusions in pig face recognition by unifying segmentation, GAN-based restoration, and lightweight classification rather than focusing solely on recognition or inpainting.
- We employ a customized YOLOv11L segmentation approach trained on pig faces with synthetic fence masks, enabling precise detection of real-farm obstructions such as metal bars that obscure critical facial cues.
- We apply inpainting technology specifically to pig face recognition for restoring occluded facial features. By targeting the challenge of fence-induced obstructions, our approach significantly enhances identification accuracy compared to baseline methods that ignore or inadequately handle such occlusions.
- We adopt EfficientNet-B2 as a resource-friendly recognition module, achieving strong identification accuracy under computational constraints. Empirical evaluations demonstrate a notable accuracy boost when occluded faces are repaired by the GAN before recognition.
- We present a newly collected and annotated dataset of pig faces frequently occluded by farm structures such as fences. This dataset captures realistic variations in lighting, pose, and environmental conditions, filling a gap in existing resources and enabling more accurate evaluations of occlusion-handling techniques in livestock identification.
- Contribution to Smart Farming and Animal Welfare. Our work provides a practical and scalable solution to the occlusion problem in pig face recognition, promoting non-invasive identification methods and supporting more efficient and ethical livestock management practices.

By addressing the “invisible barrier” of occlusions in pig face recognition, our research advances the field of smart farming technology. It demonstrates how integrating state-of-the-art computer vision techniques can overcome practical challenges in agriculture, leading to improved animal welfare and farm efficiency.

The remainder of this paper is organized as follows. Section 2 introduces the proposed PigFRIS framework, detailing methodologies for occlusion detection, image inpainting, and pig face recognition. Section 3 describes the evaluation metrics used to assess the effectiveness of our approach. Section 4 presents the experimental setup, results, and analysis, including comparisons with existing methods and ablation studies. Finally, Section 5 and Section 6 conclude the paper and outline potential directions for future research.

## 2. Materials and Methods

### 2.1. Overview of the Proposed PigFRIS System

**Figure 1:**
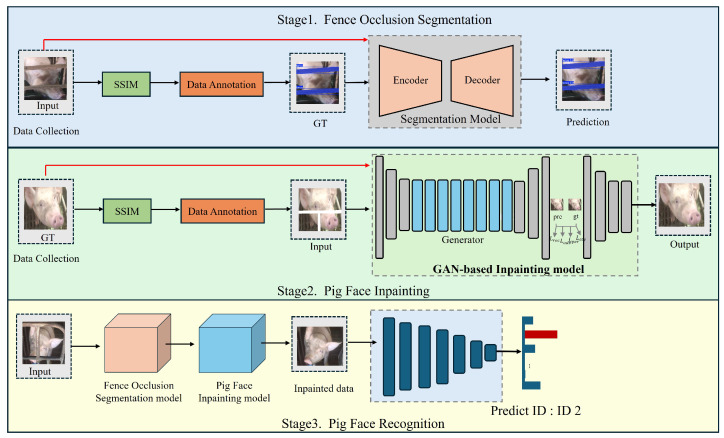
Illustration of the our proposed three-stage System PigFRIS. The top portion (Stage 1) shows how occluded pig faces are collected, annotated, and fed into the Fence Occlusion Segmentation model. The middle portion (Stage 2) depicts the GAN-based inpainting pipeline, where ground-truth (GT) images are used to train the model to restore missing facial regions. The bottom portion (Stage 3) demonstrates how inpainted pig faces are ultimately passed to a recognition network that predicts the correct pig ID, thereby overcoming the challenges posed by fence obstructions.

In this section, we detail the methodology behind our Pig Face Recognition and Inpainting System (PigFRIS) as shown in Figure 1. The system is designed to detect and restore occluded pig faces caused by metal fences, thereby improving recognition accuracy in realistic farm settings. To achieve this, PigFRIS combines three interconnected modules: (1) a supervised Fence Occlusion Segmentation model, (2) a GAN-based inpainting mechanism, and (3) a lightweight pig face recognition network. The following subsections describe the architectural overview of the system, followed by in-depth explanations of each module.

In Stage 1, to handle the commonly observed fence occlusion in farm environments, an encoder–decoder segmentation model is employed to identify and locate the occluded regions. We utilize Structural Similarity (SSIM) and manual annotations to prepare and label the pig face images, thus obtaining ground-truth masks that guide the segmentation network. Afterward, the labeled images are fed into the segmentation network during training to predict the occlusion regions. In Stage 2, once the occlusion regions are detected, the system applies a Generative Adversarial Network (GAN)-based inpainting model to fill the occluded areas. The input to the GAN consists of the original image and the occlusion masks. The generator reconstructs realistic textures for the occluded regions with the help of the discriminator, ensuring the quality of inpainting. The training process incorporates various constraints, such as perceptual loss, adversarial loss, and reconstruction loss based on the original image (ground truth), to enhance the authenticity and consistency of the inpainting results. In Stage 3, after the pig face image has been inpainted, clearer and more distinguishable facial features can be extracted for improved recognition. A classification network, whether conventional or deep learning-based, is used to extract features from the restored image, ultimately classifying the pig’s identity (e.g., “ID 2”). By combining the results of the previous two stages, Stage 3 achieves accurate recognition even in complex farm environments. By integrating these three stages, PigFRIS effectively addresses fence occlusion, improves the clarity of key facial features, and facilitates accurate and stable pig identification in real-world farm environments.

Taken as a whole, PigFRIS operates in a sequential yet tightly coupled workflow. Occlusion masks produced by the first module feed directly into the second, guiding the GAN toward precise restoration of missing features. The inpainted outputs then enter the recognition module, where even small gains in accuracy can have significant operational impact in large-scale pig farming. By addressing the systematic problem of fence-induced occlusions, our approach not only improves recognition performance but also lessens the need for invasive methods such as physical tagging or RFID implants, ultimately supporting both animal welfare and farm efficiency.

### 2.2. Dataset

**Figure 2:**
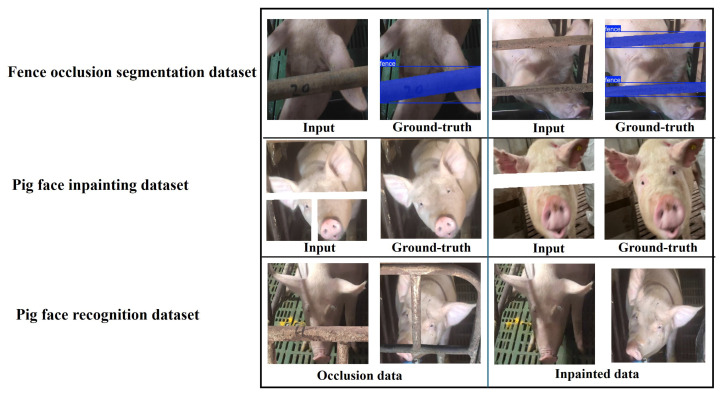
Examples of visualization from the three custom datasets used in our study. The top row showcases the Fence Occlusion Segmentation dataset, highlighting how fences partially obscure the pig’s face (input) and the corresponding ground-truth annotations. The middle row illustrates the pig face inpainting dataset, where artificially masked images (input) are paired with unoccluded ground-truth images. The bottom row shows the pig face recognition dataset, which contains both raw occlusion data and the resulting images after inpainting, enabling performance comparisons between obstructed and restored faces in the final recognition stage.

This study utilizes three distinct datasets, each corresponding to a specific task within the proposed pig face recognition pipeline. All images were collected under typical indoor farm conditions, capturing various lighting scenarios, pig poses, and occlusion patterns. Figure 2. illustrates representative samples from our three proposed datasets and Table 1. shows some details from our proposed three datasets. The first dataset targets Fence Occlusion Segmentation by pairing real occluded images with pixel-level fence annotations; the second focuses on pig face inpainting, consisting of artificially masked inputs and their unoccluded ground-truth images; and the third comprises pig face recognition data, enabling both raw occlusion and inpainted comparisons to assess performance gains in identifying individual pigs.

#### 2.2.1. Fence Occlusion Segmentation Dataset

Fence occlusions represent a significant hurdle for automated pig face recognition in indoor farming. To train our segmentation model in a supervised manner, we collected 186 images of pig faces that were actually occluded by fence bars in real farm settings. These images range from minor obstructions—where a single metal bar crosses part of the pig’s snout—to more substantial occlusions—where large portions of the face are blocked.

Each occluded image was then meticulously annotated using LabelMe. Skilled annotators outlined every visible fence segment intersecting the pig’s face at a pixel level, effectively producing a ground-truth (GT) mask for each image. This mask highlights the exact regions where the fence obscures the pig’s face, thereby instructing the model on which areas are fence-related occlusions and which belong to the pig’s facial features.

Following the annotation, the dataset was partitioned into training (60%), validation (20%), and testing (20%) subsets. Because real occlusion instances can be limited in variability, especially regarding fence orientation and lighting conditions, we augmented the training set with targeted transformations such as rotation, zoom-in, and random cropping. By diversifying how the fences appear, we increased the total size of the training set while ensuring the model is exposed to a broader spectrum of occlusion patterns.

This rigorously annotated dataset enables the YOLOv11L-based segmentation module to learn robust, fine-grained distinctions between pig facial regions and obstructing fence bars. Accurate segmentation masks are essential to the downstream inpainting step; if the fence detection is unreliable, the inpainting module risks distorting unoccluded facial features or leaving remnants of metal bars in the reconstructed images. Hence, establishing a high-quality occlusion segmentation dataset with real-world farm imagery is vital to the overall success of PigFRIS.

#### 2.2.2. Pig Face Inpainting Dataset

To address the frequent loss of critical facial details when pig faces are partially obscured by fences, we curated a dedicated inpainting dataset designed to train and evaluate supervised GAN-based inpainting methods. In a real farm environment, it is exceptionally difficult to collect perfectly matched “occluded” and “unoccluded” images of the same pig at the same pose. Therefore, our solution involves selecting pig faces with no visual obstructions as ground truth (GT) images, then artificially simulating fence-induced masks on these images to serve as the “occluded” inputs. First, a pool of 500 high-quality pig face images was collected under indoor farm conditions. Each image depicted a pig face that was not covered by any fence. These images were carefully inspected to ensure they captured varied angles, lighting conditions, and individual pig characteristics.

Next, labeling software was employed to draw fence-like shapes—replicating the typical geometry and placement of metal bars—on the otherwise unoccluded pig faces. These simulated occlusion masks formed the input data, while the original, unobstructed images were preserved as the GT. By maintaining a one-to-one pairing between artificially masked images and their unmasked counterparts, the dataset supports supervised learning, allowing the inpainting model to learn how to reconstruct occluded regions based on the GT’s intact facial features.

Following this mask generation process, the dataset was split into training (60%), validation (20%), and testing (20%) subsets. To further enrich the model’s exposure to diverse occlusion patterns, the training set was augmented with horizontal flipping and zoom-in transformations, producing an additional 1000 inpainted samples. This augmentation step ensures that the model encounters a wide spectrum of fence placements, pig facial poses, and partial occlusions—factors that mirror real-world challenges in pig farming. By training on this combination of artificially created occlusions and carefully curated GT images, the inpainting model learns to restore missing facial regions in a realistic and coherent manner, ultimately preserving essential identifying traits such as the eyes, snout, and ears. This approach balances practicality and scientific rigor. On the one hand, it respects the logistical hurdles of collecting perfectly paired real occlusion data in a farm environment; on the other, it provides a controlled, high-fidelity training set that reliably teaches the inpainting model to handle visually challenging fence obstructions. The result is a systematic, replicable methodology for generating supervised datasets that improve the completeness and accuracy of pig face images in preparation for downstream recognition tasks.

#### 2.2.3. Pig Face Recognition Dataset

Two versions of the pig face recognition dataset were curated to demonstrate performance under authentic farm conditions and to assess the efficacy of inpainting in addressing fence-induced occlusions. The first version preserves real-world occlusions captured directly from an indoor pig farm, where partial fence obstructions naturally occur. The second version applies our inpainting method to the same occluded images, reconstructing missing facial features and offering a direct comparison of how restored data influences recognition accuracy. This dataset features 20 individually tagged pigs, each assigned a unique ID and contributing approximately 50 images—resulting in around 1000 images overall. These photographs were collected over multiple sessions to capture variations in pose, lighting, and typical farm activities, reflecting everyday challenges in commercial piggery. All images were then split into training (60%), validation (20%), and testing (20%) subsets, ensuring that each pig identity was proportionally represented across the three splits.

To enhance the robustness of the training process, a range of data augmentation techniques was applied to the training set. These augmentations included horizontal flipping and zoom transformations, effectively increasing data diversity. By exposing the network to multiple angles, partial occlusions, and subtle differences in facial appearance, the model learns to generalize across the spectrum of conditions present in working farm environments. The resulting paired (occluded vs. inpainted) dataset thus enables a thorough evaluation of the pipeline’s recognition performance, both with and without restoration of fence-obscured pig faces.

### 2.3. Architecture of the Proposed PigFRIS System

#### 2.3.1. Occlusion Detection Module

Accurate detection of occlusions is paramount for the effectiveness of the PigFRIS framework. In indoor pig farming environments, metal fences frequently obstruct pigs’ faces, obscuring critical facial features essential for reliable identification. To address this, the Occlusion Detection module employs the YOLOv11 segmentation model, a state-of-the-art object detection architecture renowned for its high accuracy and real-time performance capabilities. We chose YOLOv11 because of its improved multi-scale feature extraction, balanced speed–accuracy trade-off, and enhanced segmentation performance compared to earlier YOLO versions. In particular, these characteristics allow for robust occlusion detection in challenging farm environments where high inference speed and precise segmentation are critical for subsequent inpainting and recognition tasks.

The architecture of YOLOv11 comprises several key components. At the core is the backbone network, an enhanced version of CSPDarknet, which efficiently extracts multi-scale features from input images. This backbone utilizes Cross Stage Partial (CSP) connections to facilitate better gradient flow and reduce computational redundancy, thereby enhancing feature extraction capabilities. Following the backbone, YOLOv11 incorporates a Path Aggregation Network (PANet) as the neck, which is responsible for feature fusion across different scales. PANet enhances the receptive field and improves the model’s ability to detect objects of varying sizes by effectively aggregating features from multiple layers. This multi-scale feature fusion is crucial for accurately identifying occluded regions that may vary in size and shape. The detection head of YOLOv11 is bifurcated into two primary components: the bounding box prediction head and the segmentation head. The bounding box prediction head localizes objects within the image, while the segmentation head generates pixel-wise masks for identified occlusions. The segmentation head employs lightweight convolutional layers to ensure real-time processing without compromising segmentation accuracy. Throughout the network, Leaky ReLU activation functions [22] and Batch Normalization layers [23] are utilized to accelerate training and enhance model stability.

The YOLOv11 model optimizes a composite loss function designed to balance the objectives of object detection [24] and segmentation [25]. This loss function comprises several components, including the Complete Intersection over Union (CIoU) loss [26] for bounding box optimization, defined as:

(1) $$L_{bbox}=1-\mathrm{CIoU}\left(b_{pred},b_{true}\right)$$

where $b_{pred}$ and $b_{true}$ represent the predicted and ground truth bounding boxes, respectively. To enhance classification accuracy, we utilize the cross-entropy loss:

(2) $$L_{cls}=-\sum_{c=1}^{C}y_{c}\log\left(p_{c}\right)$$

Here, *C* denotes the number of classes, $y_{c}$ is the ground truth label (one-hot encoded), and $p_{c}$ is the predicted probability for class c. For the segmentation task, Binary Cross-Entropy (BCE) loss is employed:

(3) $$L_{seg}=-\frac{1}{N}\sum_{i=1}^{N}\left[y_{i}\log\left(p_{i}\right)+\left(1-y_{i}\right)\log\left(1-p_{i}\right)\right]$$

where *N* is the number of pixels, $y_{i}$ is the ground truth mask pixel value, and $p_{i}$ is the predicted mask pixel value. The total loss function is a weighted sum of these components:

(4) $$L_{total}=\lambda_{bbox}L_{bbox}+\lambda_{cls}L_{cls}+\lambda_{seg}L_{seg}$$

where $\lambda_{bbox}$, $\lambda_{cls}$, $\lambda_{seg}$ are weight coefficients that balance the contributions of each loss component.

In the PigFRIS system, the YOLOv11 segmentation model is integrated as the core component of the Occlusion Detection module. The process begins with the input of raw pig face images captured in indoor farming environments, which are preprocessed by resizing and normalizing to meet YOLOv11’s input requirements. The model then processes these images, extracting features through the CSPDarknet backbone and fusing multi-scale features via the PANet neck. The segmentation head generates precise pixel-wise masks delineating the occluded regions caused by metal fences. These occlusion masks are subsequently utilized by the Image Inpainting module, where the Aggregated Contextual Transformations Generative Adversarial Network (AOT-GAN) reconstructs the obscured facial features. By providing accurate and detailed masks, YOLOv11 ensures that the inpainting process targets only the relevant regions, thereby preserving the integrity of the original image and facilitating high-quality restoration of missing facial features.

The choice of YOLOv11 within PigFRIS is justified by its superior performance in both accuracy and speed compared to previous versions and other object detection models. YOLOv11’s ability to perform real-time segmentation makes it highly suitable for large-scale farming operations where timely identification of occlusions is essential. Furthermore, its advanced segmentation capabilities ensure that occluded regions are precisely identified, which is critical for the subsequent image inpainting process. This integration of YOLOv11 not only enhances the accuracy of occlusion detection but also contributes to the overall efficiency and reliability of the PigFRIS system.

#### 2.3.2. Pig Face Inpainting Module

Once occlusions are detected and segmented, the next critical step is to restore the obscured facial features to facilitate accurate recognition. The Image Inpainting module utilizes the Aggregated Contextual Transformations Generative Adversarial Network (AOT-GAN), a powerful model designed for high-fidelity image reconstruction. AOT-GAN was chosen for its ability to capture both local and global contextual information through aggregated contextual transformations, resulting in more coherent and realistic inpainted regions than traditional GAN-based methods. This ensures that the reconstructed facial features seamlessly blend with the surrounding areas, preserving the integrity of the original facial structure—an essential requirement for subsequent recognition tasks.

The architecture of AOT-GAN consists of a generator and a discriminator network. The generator employs an encoder that extracts features from the masked input image, followed by an Aggregated Contextual Transformer (ACT) [27] that aggregates contextual information to fill in the missing regions. The decoder then reconstructs the inpainted image from these aggregated features. The discriminator network [28] is a convolutional neural network trained to distinguish between real and inpainted images, thereby enforcing realism in the generator’s outputs.

The AOT-GAN model is optimized using a combination of loss functions to balance reconstruction accuracy and visual realism. The primary components of the loss function include:

(5) $$L\mathrm{rec}={\left|\hat{I}-I_{gt}\right|}_{1}$$

(6) $$L\mathrm{adv}=EI_{\mathrm{gt}}\left[\log D(I_{\mathrm{gt}})\right]+E_{\hat{I}}\left[\log\left(1-D(\hat{I})\right)\right]$$

(7) $$L_{perc}=\sum_{i}\left|\right|\phi_{i}\left(\hat{I}\right)-\phi_{i}\left(I_{\mathrm{gt}}\right){\left|\right|}_{1}$$

(8) $$L_{style}=\sum_{i}\left|\right|G_{i}\left(\hat{I}\right)-G_{i}\left(I_{\mathrm{gt}}\right){\left|\right|}_{1}$$

where $|\hat{I}$ is the inpainted image, ${|}_{gt}$ is the ground truth image, *D* is the discriminator, $\phi_{i}$ represents features extracted from the i-th layer of a pre-trained network, and $G_{i}$ is the Gram matrix of features from layer I. The total loss function for the generator is a weighted sum of these components:

(9) $$L_{total}=\lambda_{rec}L_{rec}+\lambda_{adv}L_{adv}+\lambda_{perc}L_{perc}+\lambda_{style}L_{style}$$

where $\lambda_{rec}$, $\lambda_{adv}$, $\lambda_{perc}$,$\lambda_{adv}$ are hyperparameters that balance the contributions of each loss component.

In the PigFRIS framework, the AOT-GAN model serves as the core component of the Image Inpainting module. The integration process begins with the application of the occlusion masks generated by the YOLOv11 module to the raw pig face images, creating masked images where occluded regions are highlighted. These masked images, along with their corresponding masks, are then input into the AOT-GAN generator to reconstruct the obscured facial features. The generator leverages the aggregated contextual transformations to ensure that the inpainted regions are both visually realistic and structurally consistent with the non-occluded parts of the image.

The high-quality restoration provided by AOT-GAN is crucial for maintaining the integrity of the facial features, which are essential for accurate pig identification. By effectively reconstructing the occluded regions, the Image Inpainting module ensures that the Recognition module receives complete and detailed facial information, thereby enhancing the overall classification accuracy of the PigFRIS system.

#### 2.3.3. Pig Face Recognition Module

In the pig face recognition model, achieving an optimal balance between computational efficiency and classification accuracy is paramount. To this end, we employ EfficientNet-b2 as the primary recognition model within the PigFRIS framework. EfficientNet-b2 was selected due to its optimal balance between accuracy and parameter count, making it particularly suitable for real-time deployments in farm environments. By maintaining high predictive performance while minimizing computational requirements, it facilitates efficient inference under practical on-site conditions. As a member of the EfficientNet [29] family, EfficientNet-b2 leverages a compound scaling strategy that proportionally expands the network’s depth, width, and resolution. This unified approach enhances accuracy while restricting the total number of parameters relative to conventional convolutional neural networks. Consequently, EfficientNet-b2 can effectively operate on resource-limited hardware, reducing inference latency and enabling real-time implementation in pig farming scenarios.

The architecture of EfficientNet-b2 consists of a series of convolutional blocks that include mobile inverted bottleneck convolution (MBConv) layers, each augmented with squeeze-and-excitation (SE) modules to enhance feature representation by recalibrating channel-wise feature responses. The structure of EfficientNet-b2 begins with a stem block that processes the input image through a standard convolution layer, followed by a sequence of MBConv blocks that extract hierarchical features at multiple scales. Each MBConv block integrates SE modules, which adaptively recalibrate channel-wise feature maps by explicitly modeling interdependencies between channels. This mechanism allows the network to focus on the most informative features, thereby improving its discriminative capability without incurring significant computational overhead.

For the classification task, EfficientNet-b2 concludes with a fully connected layer that maps the extracted features to the number of pig identities, followed by a softmax activation function that generates probability distributions over the classes. To optimize the Recognition module, we employ the categorical cross-entropy loss function, which measures the discrepancy between the predicted probability distributions and the ground truth labels, as described in Formula (2).

Within the PigFRIS system, EfficientNet-b2 is integrated as the core component of the Recognition module. After the Occlusion Detection and Image Inpainting modules have processed the raw pig face images, the resulting inpainted images are fed into EfficientNet-b2 for classification. The model leverages its efficient architecture to rapidly process each image, extracting and emphasizing the most salient facial features necessary for distinguishing individual pigs. The use of SE modules within EfficientNet-b2 ensures that the model dynamically focuses on the most informative channels, thereby enhancing its ability to discriminate between pigs with high facial similarity. The deployment of EfficientNet-b2 in PigFRIS offers several advantages. Its compact architecture reduces the computational load, enabling real-time classification without sacrificing accuracy. This efficiency is critical for large-scale farming operations where rapid and accurate identification of individual pigs is required to monitor health, behavior, and productivity. Furthermore, the attention mechanisms embedded within EfficientNet-b2 improve the model’s robustness to variations in pose, lighting, and occlusions, ensuring consistent performance across diverse farm environments.

This multi-model approach not only improves recognition accuracy but also ensures the system’s scalability and efficiency, making PigFRIS suitable for large-scale farming operations. By leveraging the strengths of each model, PigFRIS achieves a robust and reliable pig face recognition system that enhances operational efficiency and promotes better animal welfare through non-invasive identification methods.

## 3. Evaluation Metrics

To quantitatively evaluate the performance of our classification model, we utilize classification performance metrics such as Accuracy [30], Precision [31], Recall [32], and F1-Score [33]. These metrics allow us to assess the effectiveness of our classification model comprehensively.

(10) $$\mathrm{Accurancy}=\frac{\mathrm{TP}\;+\;\mathrm{TN}}{\mathrm{TP}\;+\;\mathrm{FN}\;+\;\mathrm{TN}\;+\;\mathrm{FP}}$$

(11) $$\mathrm{Precision}=\frac{\mathrm{TP}}{\mathrm{TP}\;+\;\mathrm{FP}}$$

(12) $$\mathrm{Recall}=\frac{\mathrm{TP}}{\mathrm{TP}\;+\;\mathrm{FN}}$$

(13) $$F1-\mathrm{Score}=2\times \frac{\mathrm{precision}\times \mathrm{recall}}{\mathrm{precision}\;+\;\mathrm{recall}}$$

Accuracy provides an intuitive measure of how accurate the model is, allowing us to understand its overall outlier detection performance. The metrics are widely used in performance evaluation, with Precision indicating fewer FP (False Positives) as it approaches 1, and Recall indicating fewer FN (False Negatives) as it approaches 1. The F1-Score represents the harmonic mean between Precision and Recall, which are inversely related. A value closer to 1 indicates that the model’s performance is balanced.

We further employ FID (Frechet Inception Distance) [34] as evaluation metrics for the GAN model. These metrics are widely used in assessing the performance of generative models. FID measures the statistical difference between generated images and real images. It utilizes the Inception model to extract features from images and computes the Frechet distance (or Wasserstein-2 distance) assuming that the distributions of these features follow Gaussian distributions. The calculation formula for FID is as follows:

(14) $$\mathrm{FID}\;={\;||\mu_{r}-\mu_{g}\left|\right|}^{2}+Tr\left({\sum_{r}+\sum_{g}-2\left(\sum_{r}\sum_{g}\right)}^{1/2}\right)$$

Here, $\mu_{r}$ and $\mu_{g}$ represent the mean feature vectors of real and generated data, respectively, while $\sum_{r}$ and $\sum_{g}$ denote their respective covariance matrices. Tr denotes the trace of a matrix, and this formula represents the difference between the feature vector distributions of real and generated data. A lower FID value indicates that the quality of generated images is similar to that of real images.

SSIM(x,y) [35] is an index that measures the structural similarity between two images *x* and *y*, defined by the following formula:

(15) $$\mathrm{SSIM}\left(x,y\right)=\frac{\left(2\mu_{x}\mu_{y}+C_{1}\right)\left(2\sigma_{xy}+C_{2}\right)}{\left(\mu_{x}^{2}+\mu_{y}^{2}+C_{1}\right)\left(\sigma_{x}^{2}+\sigma_{y}^{2}+C_{2}\right)}$$

where $\mu_{x}$ and $\mu_{y}$ are the mean values of images *x* and *y*, respectively, $\sigma_{x}^{2}$ and $\sigma_{y}^{2}$ are the variance values of images *x* and *y*, respectively, and $\sigma_{xy}$ is the covariance value between images *x* and *y*. $C_{1}$ and $C_{2}$ are small constants for stabilization. The SSIM value ranges from 0 to 1, with a value closer to 1 indicating higher structural similarity between the two images.

PSNR (peak signal-to-noise ratio) [36] is another metric for measuring the similarity between two images, defined by the following formula:

(16) $$\mathrm{PSNR}\left(x,y\right)=10\log_{10}\left(\frac{{\mathrm{MAX}}^{2}}{\mathrm{MSE}(x,y)}\right)$$

where MAX is the maximum pixel value of the image, and MSE(x,y) [37] is the mean squared error between images *x* and *y*. MSE is defined as follows:

(17) $$\mathrm{MSE}\left(x,y\right)=\frac{1}{N}\sum_{i=1}^{N}{\left(x_{i}-y_{i}\right)}^{2}$$

where *N* is the total number of pixels in the image. A higher PSNR value indicates greater similarity between the two images.

MAE (Mean Absolute Error) [38] is an index that measures the absolute difference between two images, defined by the following formula:

(18) $$\mathrm{MAE}\left(x,y\right)=\frac{1}{N}\sum_{i=1}^{N}\left|x_{i}-y_{i}\right|$$

where *N* is the total number of pixels in the image. A lower MAE value indicates a smaller difference between the two images.

Thus, FID, SSIM, PSNR, and MAE are important metrics for evaluating the similarity between the generated image and the actual image. Higher SSIM and PSNR values, and lower FID and MAE values, indicate that the quality of the generated image is similar to the actual image.

## 4. Experiments

### 4.1. Fence Occlusion Segmentation

In this part of our experiment, we evaluate the effectiveness of the Fence Occlusion Segmentation module in accurately detecting and localizing occluded regions in the Fence Occlusion Segmentation dataset.

#### 4.1.1. Experimental Setup

The Fence Occlusion Segmentation experiment was conducted using the YOLOv11 Large segmentation model on a dataset specifically curated for pig face occlusions caused by fences. The input image size was consistently fixed at 640 × 640 pixels, and the dataset was split into an 80% training set and a 20% validation set. The model was trained for 200 epochs with a batch size of 16, and early stopping was applied with patience of 100 epochs to mitigate overfitting. The optimizer was automatically selected (Adam) under the Auto setting, initializing the learning rate at 0.1 and gradually reducing it by a final learning rate factor of 0.01. The momentum was set to 0.937, with a weight decay of 0.0005 to address potential overfitting. A warmup phase was employed for the first 3 epochs, starting with a warmup momentum of 0.8. In terms of loss configuration, the box loss weight was set to 7.5, the class loss weight to 0.5, and the DFL (Distribution Focal Loss) weight to 1.5. Evaluation metrics focused on segmentation accuracy, including measures such as Precision, Recall, and mAP at different IoU thresholds, ensuring a comprehensive assessment of how effectively the model identified and segmented occluded fence regions in pig face images.

#### 4.1.2. Experimental Results

In this experiment, multiple YOLO-based architectures were evaluated on a challenging occlusion segmentation dataset, where fences and other farm structures frequently obscure pig faces. The compared models include YOLO8n, YOLO8s, YOLO8m, YOLO8l, YOLO9c, YOLO11n, YOLO11s, YOLO11m, and YOLO11l. Among these, YOLO11 variants emerged as particularly effective, with YOLO11l delivering the strongest performance.

**Figure 3:**
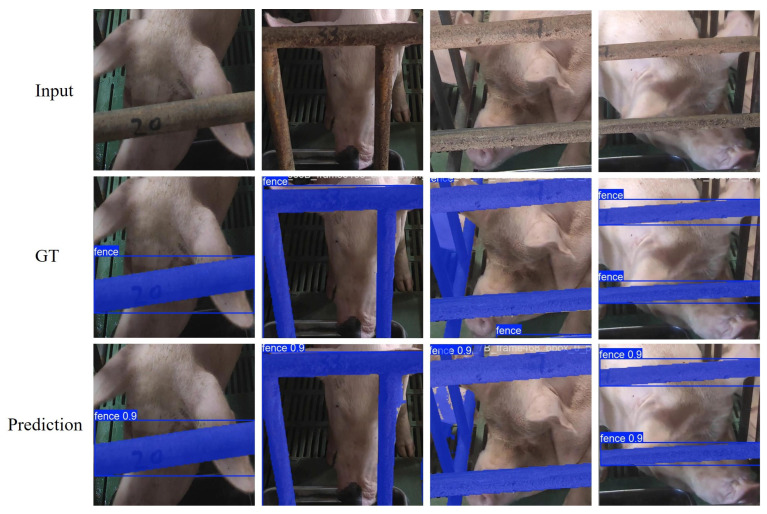
Fence Occlusion Segmentation Results using YOLOV11L model on the Testing Dataset.

Table 2 summarizes the segmentation outcomes. Notably, YOLO11l achieved an ${\mathrm{AP}}_{50}$ of 96.28%, ${\mathrm{AP}}_{75}$ of 91.9%, and ${\mathrm{AP}}_{50–95}$ of 89.48%, surpassing all other YOLO models. Additionally, it reached a Recall of 94.92%, indicating a high capacity to accurately localize occluded regions. Meanwhile, YOLO11n demonstrated a balanced performance, with a Precision of 89.49% and a Recall of 91.53%, suggesting the reliable coverage of occlusions and minimal false positives. Figure 3. presents the fence occlusion segmentation results using our customized YOLOv11L model on the testing dataset. Each column pair displays the original occluded image (top) alongside its corresponding segmentation result (bottom). The model effectively identifies and highlights fence bars (in blue) across diverse real-world occluded pig face images. Overall, these results underscore the robustness of YOLOv11l in handling complex occlusion patterns common in indoor pig farming environments. By achieving higher mAP values across diverse thresholds, YOLO11—particularly its large variant—produces more precise segmentation masks critical for subsequent inpainting. This high-quality occlusion detection not only reduces the likelihood of missed occlusions but also ensures cleaner boundaries for a more accurate restoration of hidden facial features in the ensuing stages of the PigFRIS pipeline.

### 4.2. Pig Fence Inpainting

In this phase, we compare the performance of several inpainting models to evaluate how effectively they restore occluded facial regions in the pig face inpainting dataset. Detailed descriptions of our model configurations, baseline comparisons, and evaluation metrics can be found in the subsequent subsection.

#### 4.2.1. Experimental Setup

The inpainting experiments were performed on pig face images where occluded regions were identified and masked prior to restoration. Each image was resized to 256 × 256 pixels to ensure consistent input dimensions. A comprehensive evaluation was conducted using four advanced inpainting models—DeepFillv2, AOTGAN, RFR, and TFill—to determine the most suitable model for the given application. All models were trained for 15,000 iterations, starting from pre-trained weights on the CelebA dataset as provided by the original authors. The batch size was set to 8, and the Adam optimizer was employed with a learning rate of $1\times 10^{-4}$ for both the generator and the discriminator, using Beta1 = 0.5 and Beta2 = 0.999 to ensure stable and efficient convergence. Multiple loss terms guided the model updates. An L1 Loss, weighted as 1, encouraged fidelity to the original image structure. A Style Loss, weighted as 250, preserved texture and stylistic details, while a Perceptual Loss, weighted as 0.1, ensured the restored regions aligned with high-level semantic features. To further enhance realism, an adversarial loss was incorporated at a weight of 0.01, prompting the generator to produce outputs that closely resembled authentic images.

Evaluation metrics included the Fréchet Inception Distance (FID) to assess distributional similarity, the Structural Similarity Index Measure (SSIM) and Peak Signal-to-Noise Ratio (PSNR) to quantify structural and pixel-level accuracy, and the Mean Absolute Error (MAE) to measure overall deviation from the ground truth. This combination of metrics offered a comprehensive and robust evaluation of each model’s inpainting quality. By comparing DeepFillv2, AOTGAN, RFR, and TFill under these standardized conditions, we ensured a fair assessment of performance and a well-informed decision on which inpainting model best meets the requirements of our application.

#### 4.2.2. Experimental Results

The inpainting outcomes underscore the advantages of AOTGAN in reconstructing occluded pig face images. As presented in Table 3, AOTGAN attains an FID of 51.48, outperforming other methods and suggesting a closer match to the statistical distribution of real, unobstructed images. Moreover, AOTGAN achieves an SSIM of 91.5, indicating the effective preservation of structural details and perceptual consistency in facial features. Although TFill yields marginally higher PSNR and a slightly lower MAE, these gains in pixel-level accuracy do not translate into producing globally realistic textures as effectively as AOTGAN. DeepFillv2, while offering competitive SSIM and PSNR values, exhibits a higher FID, implying less alignment with the overall realism of genuine pig faces. RFR, with substantially lower SSIM and PSNR, struggles to generate coherent restorations, revealing limitations in both fine-grained detail and global structural consistency.

Overall, AOTGAN’s balanced performance stands out. Its superior FID and SSIM, in conjunction with competitive PSNR and MAE, affirm its ability to faithfully capture intricate textures. Consequently, AOTGAN emerges as the primary inpainting choice, ensuring that restored images closely resemble high-quality, authentic data—an essential requirement for accurate pig face reconstruction.

**Figure 4:**
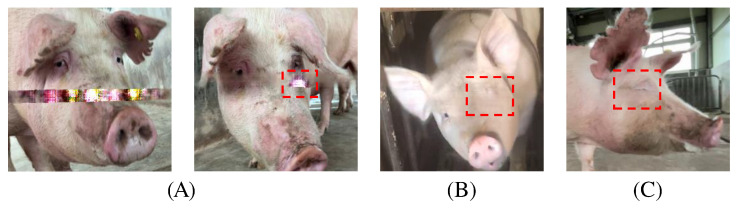
Erroneously restored images from the image inpainting experiment. (**A**) shows an overfitted inpainting result, (**B**) shows an inpainting result where one eye was removed, and (**C**) shows an inpainting result where the original open eye was restored in a closed state.

Figure 4 presents examples of failure cases observed during the inpainting experiments, illustrating that while the model generally performs effectively, certain challenging scenarios remain problematic. In (A), the model appears overfitted, producing visually inconsistent or unrealistic patterns in the restored region. In (B), a key facial feature—the pig’s eye—has been inadvertently removed during restoration, indicating difficulty in maintaining essential anatomical details. In (C), the originally open eye was restored as closed, suggesting that subtle variations in facial features are not always accurately captured. These cases highlight the limitations of the model’s current configuration and underscore the need for improved feature discrimination and context-aware reconstruction strategies. By acknowledging these shortcomings, future work can focus on refining the model to better handle complex occlusions, intricate textures, and subtle facial expressions.

**Figure 5:**
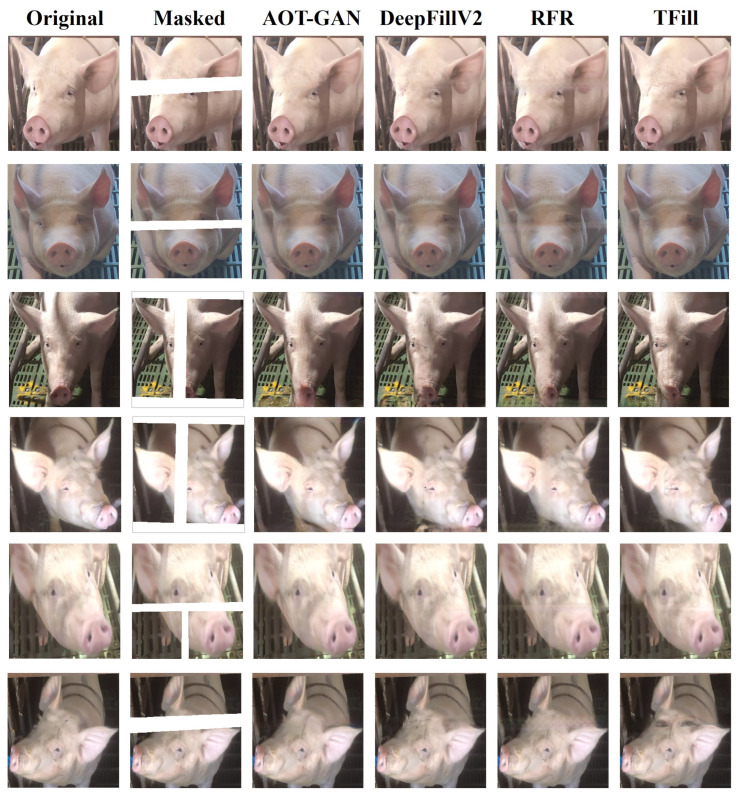
Image inpainting results of the GAN models used in the experiment.

Figure 5 displays the reconstructed pig face images produced by different inpainting models, including AOT-GAN, DeepFillv2, RFR, and TFill, alongside the original and masked inputs. While the masked images reveal substantial missing information, certain models demonstrate remarkable skill in recovering realistic facial features. AOT-GAN preserves subtle details such as the texture and contour of the pig’s ears and snout, resulting in inpainted images that closely resemble the original. DeepFillv2 also produces visually plausible restorations, though occasional artifacts or inconsistencies may appear in more complex occlusion regions. In contrast, RFR tends to struggle in maintaining coherent structure, often introducing distortions or blurred patches that reduce overall realism. TFill achieves strong pixel-level accuracy, offering slightly sharper local detail in some instances but sometimes lacks the global perceptual alignment that AOT-GAN achieves. Viewed together, these results highlight the trade-offs between different methods and emphasize that AOT-GAN consistently provides a superior balance of perceptual realism and structural integrity when restoring occluded pig faces.

### 4.3. Pig Face Classification

In this section, we compare multiple classification networks—including EfficientNet-b2—on the Pig Face Recognition dataset to accurately distinguish individual pigs using inpainted facial features. By measuring classification accuracy, inference time, and parameter efficiency, we confirm the model’s practicality in real farm scenarios. Key implementation details and evaluation metrics are provided in the following subsection.

#### 4.3.1. Experimental Setup

The pig face recognition experiments were conducted using a model initialized with ImageNet pre-trained weights. Each pig face image was resized to 256 × 256 pixels before being fed into the model. Training proceeded with a batch size of 32, and a global average pooling layer was employed to extract informative features from the convolutional backbone. To prevent overfitting, a dropout rate of 0.3 was applied, and a fully connected layer comprising 512 units and ReLU activation was introduced prior to the final classification layer. The output layer used a softmax activation function to produce class probabilities for N distinct pig identities. An Adam optimizer was employed with the learning rate set to 0.0004, and training was performed for 100 epochs. This provided sufficient time for the model to converge without incurring excessive computational expense. To comprehensively evaluate the recognition performance, Accuracy, Precision, Recall, and F1-Score were used as metrics. Accuracy measured overall correctness, Precision, and Recall assessed the model’s ability to handle class imbalances effectively, and the F1-Score provided a harmonic mean that balanced both Precision and Recall. This combination of metrics ensured a thorough understanding of the model’s classification capabilities and its practical utility in distinguishing individual pigs.

#### 4.3.2. Experimental Results

Table 4 provides a comprehensive comparison of several deep learning models on our pig face recognition dataset, showcasing the consistent and significant performance gains enabled by removing fence occlusions through the PigFRIS pipeline. Each model is evaluated in two distinct modes: training on raw, occluded images (without inpainting) versus training on images that have passed through PigFRIS’s first two stages. In these stages, a YOLOv11L-based Fence Occlusion Segmentation module initially detects and masks the fence bars covering the pig’s face, and an AOT-GAN inpainting module subsequently reconstructs the missing regions. This yields inpainted images (with inpainting), effectively restoring critical facial details around the eyes, snout, and ears for subsequent recognition.

A clear trend emerges across MobileNet-V2, MobileNet-V3, EfficientNet-B0/B1/B2, and ResNet50/101: models trained on inpainted images demonstrate higher Accuracy, Precision, Recall, and F1-Scores. This improvement underscores the benefit of accurately isolating fence bars via YOLOv11L segmentation and then reconstructing occluded areas through AOT-GAN, ensuring that classifiers receive a clearer view of key pig facial cues. Notably, both MobileNet-V2 and MobileNet-V3 exhibit marked performance jumps after occlusion removal, suggesting that even lighter-weight architectures profit from training with reconstructed data. Meanwhile, deeper ResNet networks (ResNet50 and ResNet101) also display metric boosts, indicating that enhanced facial features benefit from a broad range of model complexities. Among all tested configurations, EfficientNet-B2—employed in the third stage of PigFRIS—achieves the highest overall metrics, with Accuracy increasing from 86.22% without inpainting to 91.62% with inpainting. This strong performance reflects the synergy of EfficientNet-B2’s balanced scaling strategy with the fence segmentation and GAN-based restoration steps.

In summary, the results in Table 4 illustrate how removing fence bars and reconstructing missing features significantly elevates pig face recognition performance, irrespective of a model’s size or complexity. By integrating Fence Occlusion Segmentation (Stage 1), AOT-GAN inpainting (Stage 2), and a robust classifier (Stage 3), PigFRIS ensures that each network can focus on discriminative pig facial details. This pipeline approach validates our proposed system, demonstrating that a dedicated occlusion-removal process can substantially enhance model accuracy—particularly in architectures like EfficientNet-B2, which capitalize on richer visual information once obstructions are eliminated.

**Figure 6:**
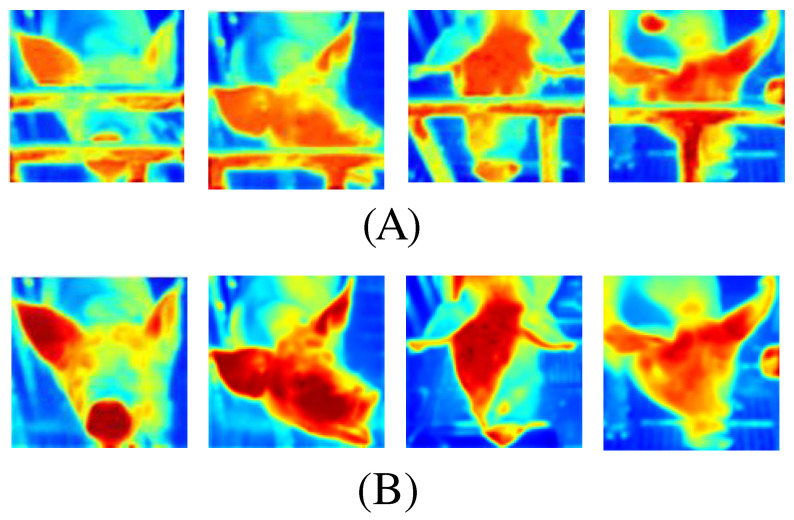
Heatmap Visualization of Occluded vs. Inpainted Pig Faces (**A**) shows heatmaps of pig faces with fence bars prominently visible as high-intensity obstructions, highlighting how occlusions disrupt the model’s ability to capture key facial cues. (**B**) displays the same images after AOT-GAN removes and reconstructs the missing regions, revealing more continuous heat distributions across the pig’s head. This inpainting step allows the network to focus on salient identifiers—such as the eyes, ears, and snout—thereby improving overall face recognition performance.

Figure 6 provides a detailed heatmap visualization that contrasts occluded pig faces (A) with their post-inpainting counterparts (B). In (A), the heatmaps clearly highlight the metal bars as regions of high intensity—typically represented in warmer colors such as red or yellow—which slice across the pig’s facial region. These high-intensity areas indicate that the segmentation model is capturing strong gradients or activation in those obstructed regions, reflecting the difficulty of extracting continuous facial features when fence occlusions are present. Conversely, (B) displays the same images after processing through our AOT-GAN inpainting module. In these visualizations, the previously occluded areas now exhibit a more homogeneous intensity distribution, with cooler colors replacing the disruptive high-intensity patterns. This shift in the heatmap signals that the inpainting process has effectively removed the metal bars and reconstructed the occluded regions, allowing the network’s attention to refocus on intrinsic facial cues such as the snout, eyes, and ears. The refined intensity gradients in (B) suggest a clearer delineation of morphological details, which ultimately contributes to improved downstream recognition performance. Overall, these heatmaps not only demonstrate the successful removal of occlusions but also provide visual evidence of how inpainting enhances feature continuity and clarity, thereby facilitating more robust pig face recognition.

**Figure 7:**
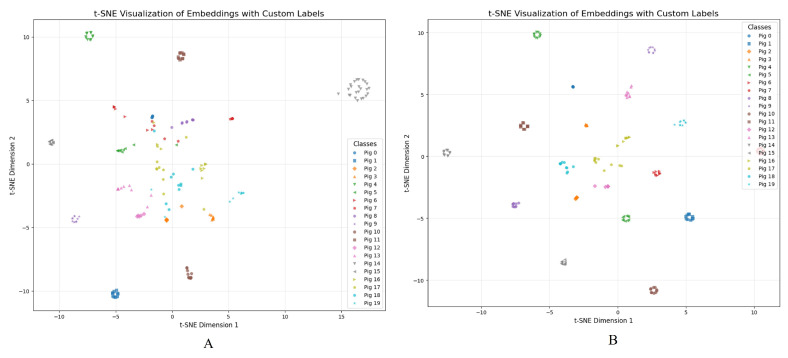
t-SNE Visualization of Occluded (**A**) vs. Inpainted (**B**) Pig Faces Using EfficientNet-B2 on Testing dataset of Pig Face Recognition Dataset.

Figure 7A displays the two-dimensional t-SNE projection of feature embeddings extracted by EfficientNet-B2 from pig faces that remain partially obscured by fence bars. Due to these occlusions, many points cluster less distinctly, indicating that key identifying cues—such as the eyes and snout—are partially missing or distorted. Figure 7B presents the same images after passing through the first two stages of PigFRIS, where YOLOv11L segments the fence regions and AOT-GAN inpaints the missing facial areas. With the obstructing bars removed and the facial features more fully restored, the embeddings form tighter, more separable clusters. This clear improvement demonstrates how accurately recovering occluded facial details before recognition empowers EfficientNet-B2 to differentiate individual pigs more effectively.

**Figure 8:**
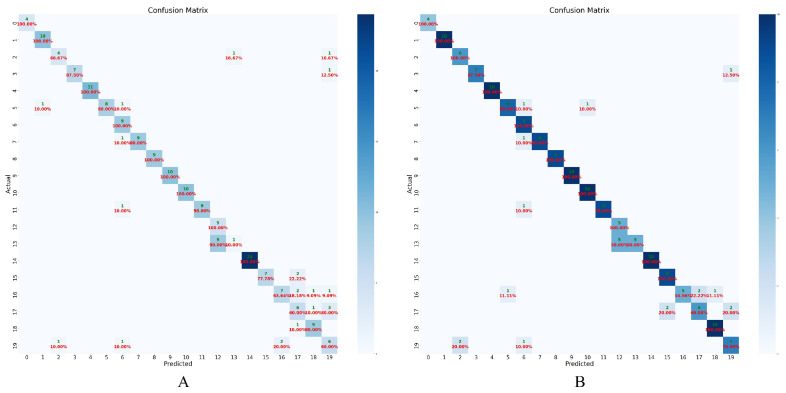
Confusion Matrix for Occluded (**A**) vs. Inpainted Pig Faces (**B**) Using EfficientNet-B2 on Testing dataset of Pig Face Recognition Dataset.

Figure 8 compares two confusion matrices reflecting EfficientNet-B2’s classification performance on (A) raw, fence-occluded pig faces and (B) faces restored via PigFRIS. In (A), a significant portion of the off-diagonal cells exhibit high values, indicating frequent misclassifications among individual pig IDs. These errors stem from missing or distorted facial cues—such as partially covered eyes or snouts—that obscure critical distinguishing traits. By contrast, (B) reveals a confusion matrix with a more pronounced diagonal and substantially lower off-diagonal counts. This improvement underscores how removing the fence and reconstructing the occluded regions enhances each pig’s unique facial signature, allowing the model to more accurately differentiate among individuals. Ultimately, the clearer diagonal trend in the inpainted scenario confirms the transformative impact of our GAN-based restoration process on overall recognition accuracy.

## 5. Discussion

The experimental findings illustrate that effectively handling occlusions is critical for improving pig face recognition performance in complex farm environments. Our occlusion segmentation module, based on the YOLOv11 model, provides highly accurate masks that are fundamental to the subsequent inpainting step. Without precise segmentation, the inpainting model would be forced to guess which regions need restoration, potentially leading to suboptimal reconstructions or the introduction of visually inconsistent artifacts. For example, in challenging lighting conditions or when fences are partially visible, the segmentation module sometimes fails to capture all occluded areas, which, in turn, causes the inpainting stage to generate blurred or distorted features. The reliable identification of occluded areas ensures that the generator focuses its efforts where it matters most—on restoring essential facial features rather than regions that are already visible.

By integrating this accurate occlusion segmentation with an advanced inpainting approach (AOT-GAN), our pipeline recovers hidden facial traits and guides the recognition model toward meaningful discriminative cues. The results confirm that when fences and other obstructions are removed at the pixel level, recognition models no longer resort to non-informative patterns for classification. Instead, they learn to rely on authentic pig facial features, ultimately increasing classification accuracy and robustness. Nevertheless, certain failure cases still persist. For instance, when the inpainting module encounters large occluded regions or complex textures—such as around the eyes or snout—it may produce reconstructions with subtle distortions or fail to preserve delicate expression nuances. These issues sometimes lead to recognition errors, particularly in instances where pigs exhibit very similar facial markings or when rapid head movements create motion blur. The presence of these failure cases highlights that even state-of-the-art segmentation and inpainting methods have limitations, suggesting that more pig-specific data and context-aware restoration algorithms are necessary to achieve consistently high-fidelity results. While leveraging pre-trained models on human-centric datasets like CelebA offers a shortcut to realistic texture generation, domain mismatches may occasionally distort the outcome. In future research, we plan to collect broader and more diverse pig face datasets, capturing a wider range of environmental conditions and facial variations. Additionally, exploring hybrid approaches that integrate other data modalities (e.g., thermal imaging or depth data) and incorporating more sophisticated generative frameworks could further improve restoration quality. Furthermore, these improvements in recognition underscore the potential impact of occlusion segmentation and inpainting on real-world livestock management systems. By ensuring that the model’s focus shifts from reliance on accidental fence patterns to genuine facial characteristics, the system gains better generalizability. In increasingly automated and data-driven farming environments, this reliability stands to benefit animal welfare monitoring, health assessment, and behavior analysis. To this end, future research should also focus on integrating the pipeline with advanced hardware solutions. Our system is designed with practical deployment in mind, being compatible with edge computing platforms and resource-constrained hardware. We plan to deploy our system using JSON Nano hardware in pig farms in Korea, aiming for real-time monitoring and efficient processing in actual field conditions. Additionally, hardware optimizations such as model quantization, pruning, and energy-efficient inference methods are promising areas to explore.

In summary, the occlusion segmentation model plays a foundational role, enabling inpainting to function optimally and thereby enhancing pig face recognition accuracy. These results underscore the importance of treating the data pipeline holistically—accurately identifying and removing occlusions at their source is as critical as the subsequent reconstruction and classification processes. This discussion not only underscores the efficacy of our proposed system but also outlines clear future research directions, particularly in improving the inpainting module to achieve more accurate pig face completion and ultimately elevate recognition performance. These avenues pave the way for more robust, adaptable, and data-efficient recognition frameworks, advancing the field of precision farming and intelligent animal husbandry.

## 6. Conclusions

In this study, we introduced an integrated pipeline that combines occlusion segmentation, inpainting, and classification to significantly enhance pig face recognition in challenging farm environments. Our approach transforms unreliable, fence-obstructed images into refined facial representations that drive more accurate and robust recognition, offering a practical solution for smart farming. Rather than relying on incidental, non-informative cues, PigFRIS focuses on authentic pig facial features, thereby improving overall recognition performance and enabling real-world applications such as livestock monitoring, health assessment, and behavior analysis. The system’s design also supports deployment on edge computing platforms, making it suitable for resource-constrained farm environments and real-time operation. We acknowledge that some limitations persist. Variations in lighting conditions, rapid animal movements, and other environmental factors can occasionally affect the precision of occlusion detection and inpainting, imposing real-time constraints on the system. To address these challenges, future research will focus on expanding the diversity of pig-specific datasets, refining our inpainting model for higher-fidelity restoration, and exploring advanced hardware optimizations—such as model quantization and pruning—for smoother, real-time performance. Overall, this work not only advances the state of the art in pig face recognition but also paves the way for more efficient, welfare-oriented smart farming solutions. By bridging advanced computer vision techniques with practical farm applications, PigFRIS establishes a solid foundation for transformative improvements in precision livestock management.

## Figures and Tables

**Table 1:** Summary of dataset augmentation, resulting sizes, and usage.

Dataset	Augmentation Strategy	Original Images	Augmented Dataset Size	Usage
Occlusion segmentation dataset	zoom + cut-out	186	558	Fence Segmentation
Pig Face Inpainting Dataset	zoom + horizontal flipping	500	1000	Occlusion Removal
Pig Face Recognition Dataset	zoom horizontal, dropout	1000	2000	Pig Face Recognition

**Table 2:** Experimental results of YOLO models on test dataset of Fence Occlusion Segmentation dataset.

Model	Precision	Recall	${\mathrm{AP}}_{50}$	${\mathrm{AP}}_{75}$	${\mathrm{AP}}_{50–95}$
YOLO8n [39]	**94.97**± 0.7	89.83 ± 1.2	95.48 ± 0.4	90.17 ± 0.1	88.62 ± 0.5
YOLO8s	87.46 ± 0.4	94.58 ± 0.2	94.87 ± 1.1	90.60 ± 0.6	84.97 ± 0.8
YOLO8m	88.71 ± 0.1	93.22 ± 0.4	96.22 ± 0.1	92.35 ± 0.9	86.86 ± 0.5
YOLO8l	90.66 ± 0.2	86.44 ± 0.4	95.78 ± 1.6	93.29 ± 0.6	84.88 ± 1.7
YOLO9c [40]	90.15 ± 1.1	93.05 ± 0.5	91.98 ± 0.1	88.82 ± 1.2	85.43 ± 0.6
YOLO11n [20]	89.49 ± 0.4	91.53 ± 1.2	93.68 ± 0.8	88.28 ± 1.3	84.21 ± 0.4
YOLO11s	88.66 ± 0.6	88.14 ± 0.2	92.41 ± 1.7	89.01 ± 0.6	85.37 ± 0.8
YOLO11m	92.04 ± 1.1	89.83 ± 0.3	92.12 ± 0.6	89.47 ± 0.4	85.01 ± 1.6
YOLO11l	87.05 ± 0.4	**94.92**± 0.7	**96.28**± 1.4	91.90 ± 1.1	**89.48**± 1.1

Note: The values that recorded the highest performance for each metric are highlighted in bold.

**Table 3:** Performance evaluation results assessed using image restoration performance metrics.

Model	FID ↓	SSIM ↑	PSNR ↑	MAE ↓
Deepfillv2 [41]	72.5 ± 0.6	90.2 ± 0.2	29.3 ± 0.2	7.2 ± 0.4
RFR [39]	80.87 ± 0.4	15.14 ± 0.9	14.21 ± 0.3	76.5 ± 1.1
TFill [19]	53.98 ± 0.4	90.9 ± 0.5	**30.34** ± 0.7	**6.3** ± 0.2
AOTGAN [21]	**51.48** ± 0.8	**91.5** ± 0.1	30.25 ± 0.5	6.6 ± 0.1

Note: The values that recorded the highest performance for each metric are highlighted in bold.

**Table 4:** Classification performance of multiple deep learning models on the test set of our pig face recognition dataset: raw (occluded) vs. PigFRIS-inpainted images.

Model	Accuracy	Precision	Recall	F1 Score
MobileNet-V2 (w/ inpainting)	**87.71**± 0.8	**87.66**± 1.1	**87.71**± 0.7	**86.27**± 0.9
MobileNet-V2 (w/o inpainting)	78.51 ± 1.0	76.96 ± 0.5	75.51 ± 1.2	73.01 ± 0.8
MobileNet-V3 (w/ inpainting)	**89.94** ± 0.6	**92.21** ± 1.3	**89.94** ± 0.8	**89.02** ± 1.2
MobileNet-V3 (w/o inpainting)	72.45 ± 0.8	83.06 ± 1.0	72.45 ± 0.9	72.86 ± 1.3
EfficientNet-B0 (w/ inpainting)	**89.39** ± 1.0	**86.52** ± 0.9	**89.39** ± 1.2	**87.11** ± 0.7
EfficientNet-B0 (w/o inpainting)	80.61 ± 1.3	86.27 ± 0.8	80.61 ± 0.6	79.80 ± 1.4
EfficientNet-B1 (w/ inpainting)	**74.30** ± 1.5	78.90 ± 0.9	**78.90** ± 1.1	**69.03** ± 1.0
EfficientNet-B1 (w/o inpainting)	71.43 ± 1.2	**81.68** ± 0.7	71.43 ± 0.8	68.38 ± 1.2
EfficientNet-B2 (w/ inpainting)	**91.62** ± 0.9	**93.22** ± 0.6	**91.62** ± 0.8	**91.44** ± 1.1
EfficientNet-B2 (w/o inpainting)	86.22 ± 1.3	87.93 ± 1.2	86.22 ± 1.0	85.88 ± 0.7
ResNet50 (w/ inpainting)	**89.39** ± 0.8	**90.83** ± 1.3	**89.39** ± 1.2	**89.05** ± 0.9
ResNet50 (w/o inpainting)	78.06 ± 1.1	83.81 ± 1.0	78.06 ± 0.7	78.03 ± 1.4
ResNet101 (w/ inpainting)	**87.15** ± 0.9	**89.52** ± 1.1	**87.15** ± 0.6	**86.14** ± 1.0
ResNet101 (w/o inpainting)	77.55 ± 1.2	80.05 ± 0.9	77.55 ± 1.3	76.46 ± 1.0

The “(w/ inpainting)” designation in the table signifies models trained on the PigFRIS-inpainting-enhanced dataset, while the “(w/o inpainting)” versions are trained on the original, occluded pig face images. Bold values indicate the best performance within the same model when comparing results with and without inpainting.

## Data Availability

The datasets generated and/or analyzed during the current study are not publicly available due to confidentiality agreements with participants but are available from the corresponding author upon reasonable request.
